# Handwashing with soap and national handwashing projects in Korea: focus on the National Handwashing Survey, 2006-2014

**DOI:** 10.4178/epih/e2015039

**Published:** 2015-08-31

**Authors:** Moo-Sik Lee, Su Jin Hong, Young-Taek Kim

**Affiliations:** 1 Department of Preventive Medicine, Konyang University College of Medicine, Daejeon, Korea; 2 Division of Infectious Disease Control, Center for Infectious Disease Control, Korea Centers for Disease Control and Prevention, Cheongju, Korea

**Keywords:** Handwashing, Attitude, Practice, Survey, Republic of Korea

## Abstract

**OBJECTIVES:**

Handwashing is the most fundamental way to prevent the spread of infectious diseases. Correct handwashing can prevent 50 to 70% of water-infections and foodborne-infections. We report the results of a fact-finding study on general handwashing attitude and practice in the Republic of Korea by analyzing habits and awareness among adults and students (grades 4 to 12) based on the 2006 to 2014 National Handwashing Surveys and observational surveys.

**METHODS:**

The awareness survey was performed by telephone interviews with adults and students in 16 municipalities and provinces sampled by quota for region, sex and age. The observational survey was performed in subway, railway, and other public restrooms in seven municipalities selected through systematic sampling.

**RESULTS:**

Adults and students washed their hands with soap/sanitizer an average of 6.6 and 5.2 times daily, respectively, in 2014, an increase and decrease compared to 2006 (4.8) and 2013 (6.8). Their average daily handwashing frequency in 2014, 9.8 and 8.3, was higher than in 2006 (7.6), but lower than in 2013 (10.3).The percentage of participants handwashing with soap after using the restroom (29.5%) has been increasing since 2009, but remain slower than in other countries (42% to 49%). The percentages of participants handwashing with water in 2014, 2013, and 2011 were 57.5%, 72.6%, and 71.4%, respectively.

**CONCLUSIONS:**

Handwashing with soap is an important national public health issue, and national projects promoting it should be given high priority. Research support is necessary to provide scientific evidence of the importance of handwashing with soap and to develop and implement evidence-based policies.

## INTRODUCTION

The United Nations Children’s Fund reports that 1.7 million children under the age of five die annually of diarrhea and pneumonia [1]. Acute respiratory infections account for 4.0 million deaths in children under five worldwide [2].

Outbreaks of water-infections and foodborne-infections in the Republic of Korea (hereafter Korea) pose serious threats, with increased incidence rates (22.0%) and affected cases (38.3%) in 2012 compared to 2011. There is an upward trend of pandemic risks associated with food poisoning, contagious eye diseases, colds, severe acute respiratory syndrome (SARS), influenza, cholera, dysentery, and meningitis [3]. Furthermore, global warming is expected to expand the ranges of tropical diseases, thus increasing the spread of weather-associated infectious diseases such as malaria, tsutsugamushi, and shigellosis [4]. Recent outbreaks of respiratory infections such as H1N1 influenza A [5], various waterborne and foodborne infectious diseases, norovirus, and multi-resistant *Staphylococcus aureus* [6] support this trend. To counteract these threats, there is a pressing need for research in related fields and development of educational programs. Handwashing is recommended as the single most effective method to prevent the spread of infectious diseases.

Handwashing with soap under running water helps prevent infectious diseases, resulting in reduced occurrences of diarrhea and respiratory infections [7]. Handwashing is easily performed, nearly free, and is highly efficacious, reducing the number of children affected with diarrhea by 30% [8] and contributing to reduced incidence of respiratory infections [9]. Handwashing may prevent the majority of infectious diseases, such as SARS, influenza, cold, cholera, dysentery, and contagious eye disease [10]. Several studies have reported reduced incidences of pneumonia, impetigo, and diarrheal diseases by over 40% to 50% through handwashing [7,11]. Handwashing has been proposed to be the most effective way to prevent spread of infection in its propagation stage, and handwashing is recommended in all stages of pandemic response to reduce the spread of respiratory viruses, including influenza viruses [12].

Soap is more effective at removing pathogens than washing with water alone [13]. According to a meta-analysis of more than 30 hand hygiene studies conducted between 1960 and 2007, handwashing contributed to reducing digestive and respiratory system diseases by 31% (95% confidence interval [CI], 19% to 42%) and 21% (95% CI, 5% to 34%), respectively. The most efficacious intervention was handwashing with soap, with no difference in efficacy between antimicrobial and non-antimicrobial soap [14].

Hand hygiene education in public facilities such as schools and kindergartens resulted in reduced risk of infection, and hand hygiene compliance in hospital settings reduced the incidence of in-hospital infections and, thus, reduced demand for hospital resources [15,16]. Worldwide, handwashing and hand hygiene education programs have high priority in public health programs, in which role models such as healthcare workers and community members (policy and government officials) encourage hand hygiene. Institutional support for funded studies and interventions should be a top-priority agenda item in order to improve hand hygiene behaviors [17].

In Korea, the National Handwashing Campaign Center launched educational programs and promotional campaigns in 2005; however, they were discontinued in 2013 due to budget issues and other problems. Despite the importance of handwashing for preventing the spread of infectious diseases, and reducing 50% to 70% of waterborne and foodborne infections, awareness and compliance among the general population remains very low, with an average number of eight handwashings per day, five of which were with soap per day (in 2011). In a 2011 handwashing fact-finding survey, 84.0% of respondents were aware that handwashing helps prevent disease, a marked increase compared with 77.6% of respondents reporting the same in 2005. In addition, most (96.3%) recognized the “correct handwashing campaign.”

The recent novel influenza outbreak increased social interest in handwashing, and most respondents (84.0%) recognized the effectiveness of handwashing for disease prevention. An increasingly large proportion of respondents had experienced handwashing educational programs or promotional advertisements (Ads)/pamphlets (57.2%). Compared to the increased awareness, however, the percentage of respondents actually washing their hands did not increase correspondingly (from 47.9% in 2005 to 57.5% in 2011).

This work investigated handwashing-related attitudes and practices of the general population and their changing trends by surveying the handwashing habits and awareness of students and adults and observing their handwashing behaviors in public restrooms. This study also compared results from the 2013-2014 National Handwashing Surveys conducted by the Korea Centers for Disease Control and Prevention (KCDC) with those of national surveys on handwashing practices and awareness conducted by the National Handwashing Campaign Center in 2006, 2008, 2009, and 2011.

## MATERIALS AND METHODS

The 2013-2014 surveys were conducted to establish statistical data necessary for setting up handwashing programs and policies related to infectious disease control planning pursuant to the Infectious Disease Control and Prevention Act.

### Awareness surveys

The 2014 awareness survey was conducted as a telephone interview of adults. A total of 5,013 adults living in 17 municipal and provincial regions sampled by quota for region, sex, and age were interviewed September 12 to 17. The student awareness survey was conducted as a face-to-face interview or online. A total of 900 students in grades 4 to 12 from 16 municipal and provincial regions nationwide were selected by quota sampling, with care taken to achieve a representative distribution of regions and grades. From September 1 to 21, face-to-face student interviews were conducted after visiting each participant at home to receive consent from their legal representative prior to interview, in compliance with Article 31 of the Act on Promotion of Information and Communication Network Utilization and Information Protection. Students in grades 8 to 12 completed an online survey with the corresponding survey agency panels from September 1 to 17.

The 2013 awareness survey of adults was conducted on September 7 by telephone interview of 1,000 male and female adults living in 16 municipal and provincial regions. The student awareness survey included 700 students in grades 4 to 12 from 16 municipal and provincial regions. From September 5 to 25, face-to-face interviews were performed on students in grades 4 to 7 after visiting each of them at home. Students in grades 8 to 12 completed online surveys from September 6 to 12.

Data from the 2006, 2008, 2009, and 2011 awareness-related surveys were extracted from the corresponding survey reports by the National Handwashing Campaign Center. The 2006 survey was performed December 2 to 3 as a telephone interview of 700 subjects aged 14 years or older selected by probability proportional to size sampling to ensure sex, age, and region-balanced distribution. The 2008 survey was performed March 10 to 20 of 600 telephone interviewees in the same manner as the 2006 survey. The same survey modalities were applied to the 2009 and 2001 surveys of 1,500 telephone interviewees in 16 municipal and provincial regions on August 1, 2009 and February 19, 2011.

The awareness survey consisted of five categories: “general handwashing habits,” “handwashing habits by situation,” “handwashing-related awareness level,” “evaluation of handwashing-related education programs,” and “evaluation of handwashing-related Ads/pamphlets.”

### Observational surveys

The 2014 handwashing observational survey was performed in subway/railway station restrooms in Seoul and six other municipalities selected by systematic sampling. During the one-week observational period (September 12 to 18), a total of 1,120 restroom users were observed and checklist completed. The 2013 observational study was performed in the same way on 840 individuals for four days (September 9 to 12). Reports from the 2006, 2008, 2009, and 2011 National Handwashing Campaign Center surveys served as the basis for the analysis. The 2006 and 2008 observational surveys, based on a checklist, were performed on 1,050 and 1,064 users, respectively, of public restrooms in Seoul and six other municipalities of two each nationwide from December 1 to 3 and March 11 to 18, respectively. The 2009 checklist-based observational survey was conducted on 5,600 public restroom users in Seoul and six other municipalities selected by systematic sampling from September 25 to October 2. The 2011 observational survey was conducted from February 28 to March 5, using the same modalities as in 2009.

The observational survey checklist contained three categories: “restroom conditions,” “task and handwashing afterwards,” and “handwashing behavior.”

## RESULTS

### Awareness survey

The average daily frequencies of handwashing with soap or hand sanitizer among adults were 6.6, 6.8, and 5.0 in 2014, 2013, and 2011, respectively. Among students, these frequencies were 5.2 and 4.7 in 2014 and 2013, respectively. The average overall daily handwashing frequencies in 2014 and 2013 were 9.8 and 10.3, and 8.3 and 7.5 for adults and students, respectively.

Most frequently, adults spent 21 seconds or longer (48.6%) per handwashing, followed by 6 to 10 seconds (23.2%). Similarly, 34.2% and 24.8% of students washed their hands for 21 seconds or longer and 6 to 10 seconds, respectively. The parts of the hand washed during handwashing were also surveyed. For adults, palms (99.7%) and backs (98.4%) both exceeded 95% in 2014, similar to 2013 (99.6% and 96.5%) and 2011 (99.3% and 96.7%), followed by fingers (84.9%) and the spaces between (79.4%).

Adult and student respondents reported similar rates of handwashing with soap (75.1% and 71.3%, respectively).

The highest proportion of adult participants reported experiencing colds (25.9%) and diarrhea (23.1%). They also reported eye infections (4.3%) and food poisoning (1.8%). The highest proportion of students reported colds (53.1%), followed by diarrhea (24.1%), eye infections (5.1%), and food poisoning (2.3%) (Table 1) [18-23].

Analysis of home handwashing habits revealed that “after bathroom use” was most common (88.0%) among adults, followed by “before eating” (71.9%) and “before preparing food” (70.2%). Among students, “before preparing food” (89.1%) was most common, followed by “after bathroom use” (86.1%) and “before eating“ (58.7%).

Among daily activities involving potential sources of infection, adult participants washed their hands more frequently “after waste disposal” (87.1%) and “after cleaning” (80.0%), and less frequently “after touching money” (18.1%) and “after coughing and sneezing” (17.6%). Student participants washed their hands more frequently “after cleaning” (79.5%) and “after waste disposal” (78.2%), and less frequently “after nosing or touching the nose” (27.3%) and “after touching money” (24.7%).

The reported frequency of handwashing among adults “after using public restrooms” was 88.0%, followed by “when returning home” (78.1%), “before eating in restaurant” (57.1%), and “use of wet towel before eating” (56.2%). The handwashing frequencies among students were “after using public restrooms” (86.2%), “when returning home” (70.6%), “before eating in a restaurant” (60.8%), and “use of wet towel before eating” (27.0%) (Table 2) [18-23].

The results of the awareness survey revealed that 90.1% and 92.3% of adult and student respondents, respectively, were aware that handwashing helps prevent infectious diseases. Adults did not wash their hands for the following reasons: “no habit yet” (31.4%), “no place to wash” (24.7%), and “cumbersome” (23.4%). Students reported “cumbersome” (35.7%) and “no habit yet” (22.9%) as the main reasons, followed by “no place to wash” (4.9%).

Most adults (59.1%) and students (65.2%) reported washing their hands more often during domestic or international pandemic outbreaks.

When asked whether public restrooms have good handwashing conditions, 29.8% of adults responded that they were “not sufficient”. The proportion of “not sufficient” responses in 2014 was lower than in 2011 (48.2%) and 2013 (31.4%); in contrast, student opinions of the conditions in public restrooms did not improve over time (48.2%, 45.3%, and 45.0% in 2011, 2013, and 2014, respectively) (Table 3) [18-23].

Results of the survey on exposure to education on correct handwashing behaviors over the previous year revealed that only 12.9% of adults reported having experienced handwashing-related education, whereas 40.6% of students had participated in handwashing education programs. Compared to 2013, adults showed a negligible increase and students a slight decrease in exposure (12.8% and 41.6%, respectively).

Adults reported an average of 2.5 handwashing-related educational experiences (n=647) in the previous year. The largest proportion (47.5%) had participated in handwashing education sessions only once, and 3.6% had experienced educational programs four times. In comparison, students (n=365) participated in an average of 1.7 handwashing educational experiences in the previous year, with “once” the most frequent response (57.0%). The most common session duration among adults and students were “<10 minutes” (51.3%) and “30 to 60 minutes”(43.6%), respectively.

The most common method of adult education was “lecture” (40.0%), followed by “audio-visual media such as video” (30.0%), “pamphlets such as community notice” (16.8%), and “practice” (12.7%). Students were most often exposed to “audio-visual media” (38.4%), “lecture” (29.0%), and “practice” (8.2%).

While 95.2% of adults and 93.2% of students felt that handwashing education was necessary in 2013, only 80.6% and 77.7%, respectively, found the handwashing education programs necessary in 2014.

More than half of the adults (57.1%; n=2,863) reported seeing Ads or pamphlets about correct handwashing. Similarly, 56.6% of students (n=509) reported seeing promotional material. Adults most frequently encountered Ads via “terrestrial TV” (47.7%), followed by “community health center and hospital plasma display panel (PDP)” (31.9%). Students mostly encountered Ads/pamphlets via “community health center and hospital PDP” (37.7%) and “terrestrial TV” (37.5%).

When asked whether the contents of the Ads/pamphlets were easy to understand, 94.1% of adults answered affirmatively, versus 3.5% who answered negatively, compared to 94.5% and 5.5% in 2013. Among students, 91.4% reported finding the contents easy to understand. Regarding their reactions to the Ads/pamphlets they had seen, 84.0% of adults reported that the Ads/pamphlets led them to consider behavioral changes including handwashing habits in 2014, a slight increase compared to 2013 (83.5%). 76.2% of students indicated that they would consider behavioral changes including handwashing after seeing the Ads/pamphlets.

Most adults (90.2%) reported having learned to wash their hands correctly after seeing the Ads/pamphlets, compared to 7.8% who did not, a slight increase from 2013 (88.5%). Nearly nine in 10 students (87.6%) answered affirmatively, vs. 9.6% who did not (Table 4) [18-23].

### Observational survey

Handwashing was observed in 29.5% of individuals after using the restroom, increases of 6.0% and 10.4% from 2013 (23.5%) and 2011 (19.1%), respectively. The highest percentage of handwashing was observed in 2009, a year with an outbreak of a novel influenza strain. Handwashing after restroom use decreased from 72.6% in 2013 to 71.4% in 2014, but handwashing with soap increased from 32.3% to 41.3%. Females washed their hands more often than males (76.6% vs. 66.2% in 2014, 81.4% vs. 63.8% in 2013, and 69.2% vs. 45.9% in 2011).

The duration of handwashing was 1 to 5 seconds in 39.0% of observed individuals, 6 to 10 seconds in 33.1%, 11 to 15 seconds for 12.9%, 16 to 20 seconds in 7.4%, and ≥21 seconds in 7.6%. The average duration increased slightly from 8 seconds in 2013 to 9 seconds in 2014.

Almost all restroom users (99.4%) turned off the faucet with their bare hands, while 0.1% used a paper towel. After handwashing, hand dryers were used most frequently (23.4%), followed by paper towels (16.3%) and personal handkerchiefs (2.9%) (Table 5) [18-23].

## DISCUSSION

Handwashing with soap is a fundamental and economical way to prevent infectious diseases, reducing the incidence of waterborne and foodborne diseases by 50% to 70% and pneumonia, impetigo, and diarrheal diseases by 40% to 50%. Handwashing is effective in preventing many infectious diseases associated with global warming, such as SARS, influenza, cold, cholera, dysentery, and contagious eye disease.

About 80% of individuals were aware that handwashing helps prevent infectious diseases. However, their handwashing habits remain weak. Moreover, although 75% reported using soap in the awareness survey, only 29.5% of restroom users did so in the observational survey, demonstrating the discrepancy between self-assessment and actual practice. Continuous fact-finding surveys are necessary to provide information on handwashing-related awareness, attitudes, and practice of the general population as the basis for handwashing campaigns.

A meta-analysis of 42 papers on handwashing and its health benefits worldwide reported that only 19% of the global population washes their hands with soap after defecation (after restroom use or diaper change), with the rate increasing from 13% and 17% in underdeveloped and developing countries to 42% to 49% in developed countries. New Zealand had the highest rate (72%), followed by the UK (52%), the Netherlands (50%), and the US (49%). The rate of handwashing with soap after restroom use in the current study (29.5%) was similar to those of Thailand (25%) and Ethiopia (22%), the least rate of handwashing with soap was Tanzania (5%).

In Korea, the KCDC, National Handwashing Campaign Center, municipal authorities, and the Ministry of Food and Drug Safety conduct and washing campaigns based on their individual objectives. However, to our knowledge, no previous study has evaluated the efficacy and validity of these programs, and only the National Handwashing Campaign Center has performed fact-finding surveys, which were recently discontinued. The survey items lacked clarity in standardization and planning, and were not systemized as a whole. The survey periods and durations were not regular, even though seasonal factors can greatly influence survey results, and sample sizes also varied between surveys. The reliability of the results is limited to simple nationwide handwashing-related statistics without the ability to adjust survey items according to previous survey results or as sociate the results with campaign programs or cooperation with other industrial sectors.

Handwashing campaigns and programs should be designed to increase awareness and good habits, and should be informed by systematic fact-finding surveys. Fact-finding surveys can assess the outcomes of handwashing surveillance and campaign programs; they can also identify points for improvement and gather respondent feedback. Well-designed programs based on this information should be used to promote handwashing with soap. These campaigns should both attract societal attention and approach individuals. Moreover, factors related to environmental conditions, understanding of campaign targets, and project background and orientation necessary for program operation must be considered, and policies should reflect these factors. Agenda frameworks should include factors useful for social marketing activities, and contain sub-frameworks aligned with the overall campaign strategies and goals.

**Figure 1.**
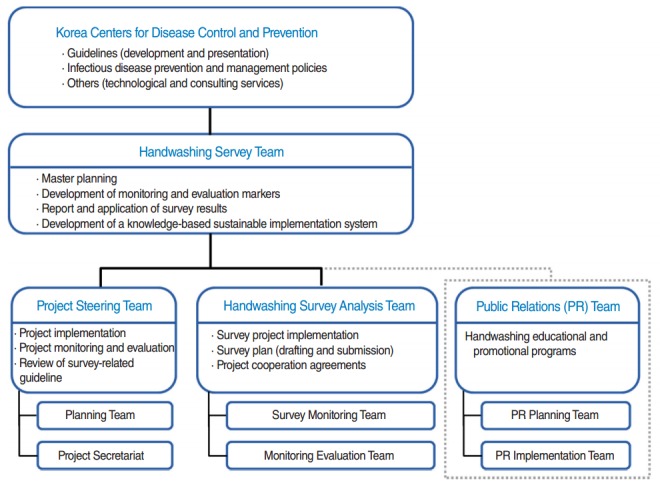
Flow chart of the handwashing fact-finding survey process. Source from Konyang University College of Medicine. National survey on handwashing with soap in Korea, 2014. Cheongju: Korea Centers for Disease Control and Prevention; 2015 [23].

Handwashing-with-soap campaigns should be recognized as an important public health issue and given high priority in implementation of public healthcare programs. To this end, an integrated collaboration system should be established among public and private sectors as well as related government departments and organizations. A strategic framework for supporting systematic fact-finding surveys and studies should be established in order to consolidate the scientific grounds to justify expansion of handwashing-with-soap campaigns (Figure 1).

This study had several limitations. First, the pre-2013 surveys had irregular survey periods and a different survey design from the post-2013 surveys in terms of participants, sample size, and survey sites, posing problems for reliable comparisons of survey results. Second, the representativeness could not be established because budget limits also sample sizes. Third, heterogeneous indicators among countries prevented observational study-based inter-county comparisons of the rates of handwashing with soap after using restrooms.

Despite these limitations, the value of this study is the nationwide monitoring and evaluation of handwashing status by municipality, which provides basic data to improve handwashing programs in individual municipalities.

## Figures and Tables

**Table 1.** Survey results related to handwashing habits

Survey items		2014		2013		2011	2009	2008	2006
		Adults	Students	Adults	Students				
Avg. handwashing w	vith soap (n/d)^1^	6.6	5.2	6.8	4.7	5.0	5.4	4.5	4.8
Sex	Male	6.1	5.5	6.6	4.5	4.5	4.7	3.9	4.2
	Female	7.1	5.1	7.3	4.8	5.5	6.0	5.0	5.4
Age (yr)	10s	-	-	-	-	3.5	3.9	3.6	3.9
	20s	6.8		6.8		5.9	5.6	5.2	4.5
	30s	7.6		7.1		6.3	6.3	5.3	5.4
	40s	6.9		7.6		5.4	5.5	4.1	5.4
	50s	3.6		6.9		4.2	5.0	4.1	4.5
	≥60	5.4		5.9					
Avg. handwashing (n/d)^1^		9.8	8.3	10.3	7.5	8.0	8.5	7.1	7.6
Sex	Male	8.4	8.9	8.7	7.5	6.8	7.0	5.7	6.0
	Female	11.2	8.0	11.7	7.5	9.1	9.9	8.5	9.2
Age	10s	-	-	-	-	5.1	5.5	4.8	5.7
	20s	8.6		8.6		7.6	7.0	7.5	6.7
	30s	10.1		10.0		9.6	9.1	8.2	8.0
	40s	10.1		11.1		8.7	8.6	7.2	8.5
	50s	10.2		10.7		7.6	9.7	7.1	7.8
	≥60	9.8		10.7					
Soap use after restroom use (%)		75.1	71.3	-	-	-	-	-	-
Avg. handwashing duration (sec)	1-5	5.8	9.1	10.5	7.6	17.7	16.6	19.7	12.9
	6-10	23.2	24.8	22.2	30.0	33.3	36.7	32.2	39.0
	11-15	7.8	14.8	8.5	13.6	15.4	15.8	16.2	18.1
	16-20	14.5	17.2	12.2	17.7	10.3	10.5	8.7	11.0
	≥21^2^	48.6	34.2	45.9	31.1	18.5	17.9	23.3	19.0
Parts of the hand washed^3^	Palms	99.7	91.9	99.6	97.4	99.3	95.6	-	-
	Backs	98.4	86.2	96.5	92.3	96.7	93.7		
	Fingers	84.9	73.4	85.3	77.1	-	-		
	Between fingers	79.4	64.6	81.1	64.3	78.9	77.7		
	Around nailbeds	73.9	53.3	75.7	58.4	-	-		
	Wrists	67.1	35.0	70.8	31.0	59.0	68.9		
	Under nails	39.9	23.7	43.9	27.3	38.2	35.8		
Diseases in the previous 6 months	Cold	25.9	53.1	24.6	52.9	43.0	25.4	45.7	38.1
	Food poisoning	1.8	2.3	2.3	2.1	1.7	1.3	1.5	1.1
	Diarrhea	23.1	24.1	25.3	31.9	-	-	-	-
	Eye infection	4.3	5.1	3.6	8.1	3.4	2.7	3.5	3.9

Avg, average.

1 Daily average over the last 7 days.

2 This includes time categories of 21-25 seconds, 26-30 seconds, and ≥31 seconds in 2013 and 2014.

3 Multiple-response item.

**Table 2.** Handwashing habits

Survey items		2014		2013		2011	2009	2008	2006
		Adults	Students	Adults	Students				
Home^1,2^	Before eating	71.9	58.7	77.5	58.7	90.3	91.9	84.5	85.9
	Before preparing food	70.2	89.1	72.5	51.7	97.3	98.2	85.3	88.7
	After bathroom use	88.0	86.1	87.7	87.3	96.2	96.7	97.3	95.0
	After nursing	68.1	-	71.2	-	81.7	91.2	87.4	85.6
	After diaper change	69.4	-	74.5	-	46.7	71.3	75.5	80.0
	After touching a pet	47.3	71.3	59.7	37.3	65.3	75.6	70.1	68.8
After contact with potential infection sources^1,2^	Money	18.1	24.7	19.1	24.8	31.3	33.5	28.5	32.2
	Cough/sneezing	17.6	36.4	17.5	19.6	28.7	26.4	24.0	21.1
	Nosing	22.1	27.3	26.1	24.2	-	-	-	-
	Waste disposal	87.1	78.2	87.5	74.0				
	Cleaning	80.0	79.5	80.2	67.2				
Public places^1,2^	Before eating in a restaurant	57.1	60.8	61.6	56.7	62.7	87.0	71.7	73.4
	After restroom use	88.0	86.2	89.0	83.7	93.7	95.7	91.0	93.3
	Before returning home	78.1	70.6	79.8	70.0	92.2	94.8	86.3	86.0
	Use of wet towel before eating	56.2	27.0	58.3	18.4	-	-	-	-

1 Values are the proportion of “always” and “generally” responses.

2 Values are the proportion of “always” and “often” responses to the 2013 and 2014 survey items.

**Table 3.** Handwashing-related awareness levels

Survey items		2014		2013		2011	2009	2008	2006
		Adults	Students	Adults	Students				
Aware of handwashing efficacy^1^		90.1	92.3	90.6	91.5	84.0	87.0	79.0	86.3
Reasons for low handwashing frequency	Cumbersome	23.4	35.7	27.5	71.2	29.2	33.7	29.5	47.1
	No place to wash	24.7	4.9	6.7	1.5	5.9	7.2	6.8	8.8
	No habit	31.4	22.9	60.5	25.8	59.7	45.2	54.5	41.2
	Others	2.4	13.8	5.3	1.5	5.2	8.9	9.1	2.9
Increased handwashing	during outbreaks	59.1	65.2	-	-	-	-	-	-
Negative opinion of handwashing environments in public restrooms^2,3^		29.8	45.0	31.4	45.3	48.2	49.7	46.0	47.1
Items necessary to improve to promote handwashing in public restrooms	Soap/sanitizer	33.2	27.1	34.5	30.1	-	-	-	-
	Clean environment	23.3	27.8	28.4	39.7				
	Hand dryer/paper towel	16.6	12.8	15.8	13.3				
	Washing basin repair	6.6	7.3	8.3	10.1				
	Education/public relations (guidelines)	3.5	1.6	4.7	2.0				
	Hot water supply	2.3	5.0	2.5	4.4				

1 Values are the proportion of “very helpful” and “fairly helpful” responses.

2 Values are the proportion of “very poor” and “fairly poor” responses in the 2009 and 2011 surveys.

3 Values are the proportion of “not sufficient at all” and “not sufficient” responses in the 2013 and 2014 surveys.

**Table 4.** Evaluation of handwashing-related educations and Ads/pamphlets

Survey items		2014		2013		2011	2009	2008	2006
		Adults	Students	Adults	Students				
Exposure to education/promotion		-	-	-	-	57.2	48.5	27.2	17.4
Exposure to education		12.9	40.6	12.8	41.6	-	-	-	-
Experience with educating children		54.4	-	71.0	-	58.9	66.8	70.4	66.8
Exposure to education from parents		-	50.4	-	-	-	-	-	-
Education frequency over the past year (n)	1	47.5	57.0	45.6	54.6	-	-	-	-
	2	26.3	30.4	33.8	23.0				
	3	11.7	6.6	7.3	13.4				
	4	3.6	1.6	6.6	1.7				
	≥5	11.0	4.4	6.8	7.2				
Average educational session duration (min)	<10	51.3	13.7	46.6	18.2	-	-	-	-
	10-30	30.7	34.8	33.3	40.5				
	30-60	11.1	43.6	12.7	37.8				
	≥60	5.2	5.5	6.0	3.4				
Educational method	Lecture	40.0	29.0	43.0	35.7	-	-	-	-
	Audio-visual	30.0	38.4	35.6	32.6				
	Leaflet	16.8	23.0	12.7	24.7				
	Practice	12.7	8.2	8.7	6.5				
Awareness of the necessity of education		80.6	77.7	95.2	93.2	-	-	-	-
Exposure to handwashing Ads		57.1	56.6	56.4	56.1	-	-	-	-
Ads medium	Terrestrial TV	47.7	37.5	47.0	46.8	-	-	-	-
	Hospital PDP	31.9	37.7	29.0	41.2				
	Internet	7.8	16.7	8.2	18.6				
	Bus	4.8	9.6	3.6	15.8				
	Subway	10.4	6.5	8.0	10.7				
	Cable TV	5.5	7.7	4.6	6.4				
	Outdoor LED display	7.2	8.1	9.9	6.1				
Understanding of Ads/pamphlets1		94.1	91.4	94.5	95.2	-	-	-	-
Change of attitude and awareness after exposure to Ads/pamphlets^1^		84.0	76.2	83.5	80.1	-	-	-	-
Consideration of handwashing method after exposure to Ads/pamphlet^1^		90.2	87.6	88.5	90.1	-	-	-	-

Ads, advertisements; PDP, plasma display panel; LED, light-emitting diode.

1 Values reflect the responses “very much so” and “generally so”.

**Table 5.** Handwashing observational survey results

Survey item and category		2014	2013	2011	2009	2008	2006
Handwashing with soap after using public restrooms		29.5	23.5	19.1	35.6	18.6	17.0
Use of soap while washing hands		41.3	32.3	33.2	47.6	31.1	30.0
Handwashing after using public restrooms		71.4	72.6	57.5	74.7	60.1	63.5
Sex	Male	66.2	63.8	45.9	64.7	53.6	-
	Female	76.6	81.4	69.2	84.8	66.5	
Age	10s^1^	68.2	67.3	53.7	67.0	65.4	-
	20s	76.7	75.9	65.9	80.4	64.7	
	30s	74.5	76.4	60.5	74.	63.4	
	40s	73.6	72.2	52.9	72.9	52.5	
	≥50s	62.7	64.9	40.7	65.5	54.0	
Region	Seoul	78.8	68.3	51.0	80.8	57.9	-
	Incheon	73.1	71.7	57.3	80.6	50.7	
	Busan	63.1	67.5	46.6	63.1	61.2	
	Daegu	68.1	75.0	56.8	73.6	65.1	
	Kwangju	62.5	75.8	50.4	70.0	67.1	
	Daejeon	79.4	75.0	57.3	76.9	57.2	
	Ulsan	75.0	75.0	83.5	78.1	61.2	
Average handwashing duration (sec)	1-5	39.0	46.6	39.3	27.7	40.5	45.4
	6-10	33.1	29.0	28.8	28.2	32.9	31.6
	11-15	12.9	12.1	17.8	19.7	16.9	13.6
	16-20	7.4	7.5	8.8	14.9	6.6	5.4
	≥21	7.6	4.8	5.3	9.5	3.1	3.9
Turning off the faucet	Bare hands	99.4	99.2	95.7	98.8	43.8	58.2
	Paper towel	0.1	-	3.2	0.5	0.2	-
	Automatic^2^	0.3	0.8	1.1	0.7	56.0	41.8
Drying after handwashing	Paper towel	16.3	14.1	20.4	22.2	49.9	54.3
	Hand towel for multiple use	-	-	-	-	0.2	-
	Hand dryer	23.4	23.9	30.4	30.0	25.8	15.7
	Personal handkerchief	2.9	3.1	3.5	3.4	1.4	2.8
	Not drying^3^	52.0	57.9	41.0	42.4	22.7	27.1

1 In the 2009 and 2011 behavior surveys, “10s” was replaced with “≤10”.

2 In the 2009 and 2011 behavior surveys, “automatic” was replaced with “not turning off the faucet”.

3 In the 2009 and 2011 behavior surveys, “not drying” was replaced with “letting dry/others”.
