# Supplementary material for: Handwashing with soap and national handwashing projects in Korea: focus on the National Handwashing Survey, 2006-2014
Source: Epidemiol Health. 2015 Aug 31;37:e2015039. doi: 10.4178/epih/e2015039 (PMC4652062; doi:10.4178/epih/e2015039)
Supplement: Supplementary file 1 [file epih-37-e2015039-supplementary.pdf]

Handwashing with Soap and National Handwashing Projects in Korea : Focus on National Handwashing Survey, 2006~2014

Moo-Sik Lee[1*], SuJin Hong[1], Young-Taek Kim[2]

Department of Preventive Medicine, College of Medicine, Konyang University[1]

Department of Korea Center for Disease Control and Prevention[2]

[*] Corresponding Author : Moo-Sik Lee(Konyang Univ.)

Tel: +82-42-600-6404 email: mslee@konyang.ac.kr

Abstract

OBJECTIVES

손씻기는 감염병 예방을 위한 가장 기초적인 방법으로 수인성 및 식품매개감염병의 약 50~70%가 손씻기를 통해서 예방이 가능하다. 이 글은 '2006~2014년도 손씻기 실태조사'의 결과를 비교·보고함으로써, 성인남녀 및 초·중·고등학생을 대상으로 평소 손씻기 실태 및 인식수준, 공공화장실 이용 시 행동 등을 파악하여, 비누로 손씻기와 관련한 국민 태도 및 실태를 살펴보고자 하였다.

METHODS

인식도 조사는 지역, 성, 연령별 비례할당추출(Quota Sampling)을 통하여 전국16개 시도에 거주 중인 성인 및 초·중·고등학생 대상으로 전화조사를 통해 진행하였다. 관찰조사는 계통추출을 통하여 서울 및 6대 광역시 지하철역(기차역) 또는 공공화장실의 이용객을 대상으로 관찰조사를 통해 진행되었다.

RESULTS

평소 손을 씻을 때 비누(손세정제)를 사용하는 빈도를 조사한 결과, 2014년도 성인의 경우 하루 평균 6.6회로 조사되었으며, 학생의 경우 하루 평균 5.2회로 나타났다. 2006년도 조사결과인 4.8회보다 증가하였으며, 2013년도 조사결과(평균 6.8회)보단 감소하였다. 하루 평균 손씻기 빈도를 살펴본 결과, 성인들의 경우 9.8회로 나타났으며, 학생들의 평상시 손씻기 빈도는 하루 평균 8.3회로 조사되었다. 비누사용빈도와 마찬가지로, 2006년도 평균 7.6회보다 증가하였으며, 2013년도 평균 10.3회보다 감소하였다.

관찰조사 결과, 2014년도에는 공공화장실 이용 후 비누로 손씻기율이 29.5%로 2009년도를 제외하고 꾸준히 증가하고 있고 있지만, 선진국의 42%에서 49%에 비해 낮은 실천율을 보이고 있다. 공공화장실 이용 후 손씻기율은 2014년도 71.4%로 조사되었으며 2013년도 72.6%, 2011년도 57.5%로 매년 차이가 존재하였다.

CONCLUSIONS

비누로 손씻기 사업을 국가의 중요한 공중보건문제로 인식하고 보건의료사업에서 기본적이고, 우선순위 사업에 설정토록 해야 하며 체계적인 손씻기 실태조사 체계를 구축하여 과학적 근거를 확보하고 근거 기반의 효율적인 정책을 개발·실행하기 위한 연구 개발을 지원하는 전략 틀이 필요하다.

Key Words : 비누로 손씻기, 손씻기 실태, 인식

I. Introduction

UNICEF는 연간 약 400만 명의 5세 이하 개발도상국 어린이들이 설사질환과 폐렴으로 사망하고 있다고 보고했다[1]. 또한, 주요 급성 호흡기 감염질환으로(Acute Respiratory Infections, ARI) 연간 약 180만의 5세 미만 어린이가 사망하고 있다. 이로 인해 약 1조 300억 원 손실이 발생하고 있다[2].

우리나라에서는 2012년 수인성·식품매개질환 유행 발생이 2011년 동기간에 비해 발생 건수는 33.3%, 환례 수는 58.5% 증가하였고 현재 식중독, 유행성 눈병, 감기, 중증급성호흡기증후군(SARS), 독감, 콜레라, 이질, 수막염 등의 감염병의 발생 및 확산의 위험이 증가하고 있다[3]. 또한, 지구온난화로 인한 열대 감염병의 확산으로 앞으로는 말라리아, 쯔쯔가무시, 세균성이질 등의 기후관련 감염질환이 더욱 더 증가할 것으로 예상되고 있고[4] 최근 신종인플루엔자[5]등 호흡기 감염병, 수인성 및 식품매개성 감염병, Norovirus, MRSA[6] 등이 지속적으로 발생하고 있다. 따라서 관련 분야에 대한 적극적인 연구 및 홍보방안 개발이 요구되고 있으며, 예방수칙 준수 및 그 중 하나인 손씻기 실천을 권장하고 있다.

비누로 손씻기는 설사와 호흡기 질환의 감소에 영향을 주는 것[7]으로 대표적인 감염병 예방수칙이다. 손씻기는 간단하고 저렴하며, 손씻기를 통해 설사 환아의 30%를 감소시키고[8] 호흡기감염병의 감소와 관련있다[9]. 사스, 인플루엔자, 감기, 콜레라, 이질, 유행성 눈병 등 대부분의 전염병이 손씻기로 예방 가능하며[10] 손씻기로 폐렴, 농가진, 설사질환의 40~50% 이상 감소를 보고하였다[7][11]. 신종인플루엔자 대응단계별 손씻기 효과에 대한 전문가 의견수렴 결과, 신종인플루엔자 확산단계에서 모든 환자들 간 감염 전파를 막는 방법 가운데 손씻기가 중요한 대책으로 제시되고 있으며, 손씻기는 인플루엔자를 포함한 호흡기 바이러스의 전파를 줄이는데 효과적이라고 제시되고 있어 모든 단계에서 권고되어진다[12].

또한, 물로 손을 씻는 것보다 비누를 사용하는 손씻기가 세균을 효과적으로 제거한다[13]. 1960~2007년 사이의 손 위생(hand-hygiene trials) 연구에 대한 메타분석(총 30편)결과, 손씻기는 소화기질환의 31% 감소(95% CI : 19~42%), 호흡기질환 21%(95% CI : 5~34%) 감소를 보였다. 가장 효과적인 중재는 비항균 비누를 이용한 손 위생 교육이였으며, 항균비누와 비항균비누 사이에는 효과 차이가 없었고 비누를 사용하는 것이 효과적이었다[14].

학교와 어린이집 등 기관에서는 감염위험감소 효과를 보였고, 병원에서는 병원내 감염의 감소, 병원자원의 절약 등을 가져오는 효과를 보였다[15][16]. 세계적으로 손씻기 및 손 위생 사업은 공중보건 영역에서 우선순위가 높은 사업으로 정책관계자, 신생아실, 학교, 공무원 등 보건의료종사자

와 지역사회 구성원으로 구성된 롤 모델(role model)이 필요하며 아울러 손 위생행동의 개선을 위한 지원과 우선순위 사업으로 자리 매김 및 기금 연구, 중재 등이 필요하다[17].

국내에서는 2005년부터 '범국민손씻기운동본부'를 통해 손씻기 사업을 시작하였으나, 예산 미흡 등 문제로 사업이 중단되었다. 손씻기는 감염병 예방을 위한 가장 기초적인 방법으로 수인성 및 식품매개감염병의 약 50~70%는 손씻기를 통해서 예방이 가능하지만, 아직까지 국민들의 인식과 실천이 일일 손씻기 빈도 8회, 비누사용빈도 5회, 비누 사용률 63.2% 수준(2011년)으로 미흡한 상황이다.

2005년과 2011년에 시행된 손씻기 실태조사 보고서에 의하면 손씻기의 질병 예방효과에 대해서 '도움이 된다.'는 응답이 2005년 77.6%에서 2011년 84.0%로 증가되었으며 '올바른 손씻기 운동'에 대해 거의 대다수(96.3%)가 알고 있는 것으로 조사되었다.

2009년 전 세계적으로 유행하였던 신종플루 이후 손씻기에 대한 사회적 관심이 더욱 증가하였으며 2009년 이후 손씻기를 통한 질병 예방 효과에 대해 국민 대부분(84.0%)이 공감하고 있으며, 손씻기 교육 및 홍보물에 대한 경험(57.2%)도 지속적으로 증가하고 있으나 이러한 인식의 증가에도 불구하고, 실제로 손을 씻는 사람의 비율은 2005년 47.9%, 2011년 57.5%로 비율 자체는 다소 증가하였지만, 84.0%에 달하는 인식도에 비해서 실천은 아직 많이 부족한 실태이다.

이 글에서는 질병관리본부의'2013~2014년도 손씻기 실태조사' 및 범국민손씻기운동본부에서 진행되었던 '2006, 2008, 2009, 2011년도 손씻기 실태 및 대국민 인식도 조사'의 결과를 비교 보고함으로써, 초·중·고등학생 및 성인 남녀를 대상으로 평소 손씻기 실태 및 인식수준, 공중화장실 이용 시 행동 등을 파악하여, 손씻기와 관련한 국민 태도 및 실태를 살펴보고자 한다.

II. Subjects and methods

2013~2014년도 조사는『감염병의 예방 및 관리에 관한 법률』에 근거하여 비누로 손씻기 실태를 파악하여, 감염병관리사업 및 정책 수립과 평가에 필요한 통계자료를 산출하기 위해 실시했다.

1. 인식도조사

2014년도 손씻기 인식도조사(성인)는 지역, 성, 연령별 비례할당추출을 통하여 전국 17개 시도에 거주 중인 만 19세 이상 성인 남녀 5,013명을 대상으로 전화면접조사로 9월 12일부터 17일까지 실시하였다. 손씻기 인식도조사(학생)는 지역, 학년별 비례할당추출을 통하여 전국 16개 시도에 거주 중인 초4학년~고3학년 학생 900명을 대상으로 시행했다. 초등학생 4~6학년과 중학생 1학년의 경우, 정보통신망 이용촉진 및 정보보호 등에 관한 법률 제 31조(법정대리인의 권리)를 준수하기 위해 직접 법정대리인의 동의를 받기위해 방문조사로 9월 1일부터 21일까지 진행하였으며 중학생 2~3학년과 고등학생 1~3학년의 경우에는 리서치조사기관의 기존 패널들을 대상으로 웹조사를 9월 1일부터 17일까지 시행하였다.

2013년도 손씻기 인식도조사(성인)는 지역, 성, 연령별 비례할당추출을 통하여 전국 16개 시도에 거주 중인 만 19세 이상 성인 남녀 1,000명을 대상으로 전화면접조사로 9월 7일 실시하였다. 손씻기 인식도조사(학생)는 지역, 학년별 비례할당추출을 통하여 전국 16개 시도에 거주 중인 초4학년~고3학년 학생 700명을 대상으로 시행했다. 초등학생 4~6학년과 중학생 1학년의 경우, 방문조사로 9월 5일부터 25일까지 진행하였으며 중학생 2~3학년과 고등학생 1~3학년의 경우에는 리서치조사기관의 기존 패널들을 대상으로 웹조사를 9월 6일부터 12일까지 시행하였다.

2006년, 2008년, 2009년, 2011년 결과는 범국민손씻기운동본부에서 시행한 '손씻기 실태 및 대국민 인식도 조사 결과보고서'를 참고로 하였으며 2006년 '손씻기 실태 및 대국민 인식도 조사'는 성/연령/지역별 크기비례확률추출을 통하여 전국 14세 이상 일반국민 700명을 대상으로 전화조사를 12월 2일부터 3일까지 실시하였다. 2008년 '손씻기 실태 및 대국민 인식도 조사'의 경우, 성/연령/지역별 크기비례확률추출을 통하여 전국 14세 이상 일반국민 600명을 대상으로 전화조사를 3월 10일부터 20일까지 실시하였다. 2009년 '손씻기 실태 및 대국민 인식도 조사'는 지역/성/연령별 비례할당추출을 통하여 전국 16개 시도에 거주 중인 만 14세 이상 일반국민 1,500명을 대상으로 전화면접조사로 8월 1일에 실시하였다. 2011년은 2009년과 동일한 방법으로 2월 19일에 시행하였다.

인식도조사는 '평소 손씻기 실태', '상황별 손씻기 습관', '손씻기 관련 인식수준', '손씻기 관련 교육에 대

한 평가', '손씻기 관련 광고/홍보에 대한 평가' 총 5가지 항목으로 구분된다.

2. 관찰조사

2014년도 손씻기 관찰조사는 계통추출을 통하여 서울 및 6대 광역시 지하철역(기차역) 화장실 이용자 총 1,120명을 대상으로 구조화된 Check-List를 이용한 관찰조사를 9월 12일부터 18일까지 실시하였다. 2013년도 조사는 계통추출을 통하여 서울 및 6대 광역시 지하철역(기차역) 화장실 이용자 총 840명을 대상으로 구조화된 Check-List를 이용한 관찰조사를 9월 9일부터 12일까지 실시하였다. 2006년, 2008년, 2009년, 2011년 결과는 범국민손씻기운동본부에서 시행한 '손씻기 실태 및 대국민 인식도 조사 결과보고서'를 참고로 하였으며 2006년 '손씻기 실태 및 대국민 인식도 조사-관찰조사'는 전국 7대 도시의 2지점을 선정하여 공공화장실 이용자 총 1,050명을 대상으로 Check-list를 이용한 관찰조사를 12월 1일부터 3일까지 실시하였다. 2008년 '손씻기 실태 및 대국민 인식도 조사-관찰조사'는 전국 7대 도시의 2지점을 선정하여 공공화장실 이용자 총 1,064명을 대상으로 Check-list를 이용한 관찰조사를 3월 11일부터 18일까지 실시하였다. 2009년 '손씻기 실태 및 대국민 인식도 조사-관찰조사'는 계통추출을 통하여 서울 및 6대 광역시 주요 지점을 선정하여 공공화장실 이용자 총 5,600명을 대상으로 Check-list를 이용한 관찰조사를 9월 25일부터 10월 2일까지 실시하였다. 2011년은 2009년과 동일한 방법으로 2월 28일부터 3월 5일까지 시행하였다.

계절과 유행성 질환에 따라 손씻기 횟수가 달라지므로 기상변화 및 황사 등 환경변화의 영향이 적은 9월 초순에 시행을 결정했고, 손씻기 관찰조사는 '화장실 상황', '화장실 용변 종류 및 손씻기 여부', '손씻기 행동' 총 3가지 항목으로 나뉜다.

III. Results

1. 인식도조사

평소 손을 씻을 때 비누 또는 손세정제를 사용하는 빈도를 조사한 결과, 성인은 2014년도 평균 6.6회로 조사되었으며, 2013년도에는 평균 6.8회, 2011년도에는 평균 5.0회로 조사되었다. 만 11~18세 학생의 경우, 2014년도 평균 5.2회, 2013년도 평균 4.7회로 조사되었다. 하루 평균 손씻기 빈도를 살펴본 결과, 성인은 2014년 평균 9.8회, 2013년 평균 10.3회, 2011년 평균 8.0회로 나타났으며 학생은 2014년 평균 8.3회, 2013년 평균 7.5회로 조사되었다.

평소 손을 한번 씻는데 소요되는 시간을 살펴본 결과, 성인의 경우 '21초 이상'(48.6%)과 '6~10초'(23.2%)의 응답이 가장 높게 나타났으며, 학생들은 한번 손 씻는데 소요되는 시간으로 '21초 이상'(34.2%)과'6~10초'(24.8%)의 응답으로 유사하였다. 손 씻는데 소요되는 시간에 대한 시계열 분석 결과, 성인과 학생 모두 전반적으로 손을 씻는 시간이 크게 늘어난 양상이며 구체적으로 살펴보면, 성인의 경우 2013년에 비해 '1~5초'(↓4.7%), '11~15초'(↓0.7%),에 대한 응답은 소폭 하락한 반면, '6~10초', '16~20초','21초 이상' 씻는다는 응답은 상승한 것으로 조사되었다. 또한, 성인의 경우 2011년에 비해 '1~5초'(↓11.9%), '11~15초'(↓10.1%), '6~10초'(↓7.6%)에 대한 응답은 하락한 반면,'16~20초','21초 이상' 씻는다는 응답은 상승한 것으로 조사되었다.

손을 씻을 때 일반적으로 손의 어느 부위까지 씻는지에 대해서 조사한 결과, 성인의 경우 '손바닥'(99.7%)과 '손등'(98.4%)을 씻는다는 비율이 95% 이상으로 높게 나타났으며, 이는 2013년도에 '손바닥'(99.6%)과 '손등'(96.5%), 2011년도에 '손바닥'(99.3%)과 '손등'(96.7%)로 나타난 것과 유사하다. 다음으로 '손가락'(84.9%), '손가락 사이'(79.4%) 등의 순서로 나타났다. '손톱 밑'을 씻는 다는 응답은 39.9%로 다른 부위에 비해 상대적으로 낮게 조사되었다. 이는 '손톱 밑'을 씻는다는 응답에 2013년도에 43.9%, 2011년도에 38.2%로 다른부위에 비해 상대적으로 낮게 조사된 것과 동일하였다. 학생의 경우에도 성인과 비슷한 결과를 보였으며 성인과 학생 모두 거의 대부분의 계층에서 '손바닥'부위를 씻는다는 응답이 2014년도, 2013년도, 2011년도 모두 90% 이상으로 높게 조사되었다. 화장실을 이용한 후 비누를 사용하는지 여부에 대해 조사한 결과, 성인의 경우 '비누를 사용한다'는 응답이 75.1%로 높게 나타났으며, 학생의 경우에도 화장실 이용 후 비누를 사용한다는 응답이 71.3%로 성인과 비슷한 경향을 보인다.

최근 6개월 이내에 걸린 질환에 대해 조사한 결과, 만 19세 이상 성인의 경우 ‘설사’(23.1%)와 ‘감기’(25.9%)에 걸린 적이 있다는 응답이 가장 높게 나타났으며, ‘눈병’은 4.3%, ‘식중독’은 1.8%가 걸린 경험이 있는 것으로 조사되었다. 만11세부터 만18세 사이의 학생의 경우에는 ‘감기’에 걸린 적이 있다는 응답이 53.1%로 가장 높았으며, 다음으로 ‘설사’(24.1%). ‘눈병’(5.1%), ‘식중독’(2.3%) 순으로 나타났다. ‘설사’에 걸린 경험은 손씻기 빈도가 낮을수록(3회 이하 26.6% > 11회 이상 20.3%) 높은 특징을 보였다.

표 1. 평소 손씻기 실태조사 결과 단위: %

		2014년		2013년		2011년	2009년	2008년	2006년
		성인	학생	성인	학생				
조사대상		19세 이상	11~18세	19세 이상	11~18세	14세 이상	14세 이상	14세 이상	14세 이상
최근 1주일 동안 평균적으로 비누사용빈도(회/일)		6.6	5.2	6.8	4.7	5.0	5.4	4.5	4.8
성별	남성	6.1	5.5	6.6	4.5	4.5	4.7	3.9	4.2
	여성	7.1	5.1	7.3	4.8	5.5	6.0	5.0	5.4
연령	10대	-	-	-	-	3.5	3.9	3.6	3.9
	20대	6.8		6.8		5.9	5.6	5.2	4.5
	30대	7.6		7.1		6.3	6.3	5.3	5.4
	40대	6.9		7.6		5.4	5.5	4.1	5.4
	50대	3.6		6.9		4.2	5.0	4.1	4.5
	60대 이상	5.4		5.9					
최근 1주일 동안 평균적으로 손 씻은 빈도(회/일)		9.8	8.3	10.3	7.5	8.0	8.5	7.1	7.6
성별	남성	8.4	8.9	8.7	7.5	6.8	7.0	5.7	6.0
	여성	11.2	8.0	11.7	7.5	9.1	9.9	8.5	9.2
연령	10대	-	-	-	-	5.1	5.5	4.8	5.7
	20대	8.6		8.6		7.6	7.0	7.5	6.7
	30대	10.1		10.0		9.6	9.1	8.2	8.0
	40대	10.1		11.1		8.7	8.6	7.2	8.5
	50대	10.2		10.7		7.6	9.7	7.1	7.8
	60대 이상	9.8		10.7					

화장실 사용 후 비누 사용율		75.1	71.3	-	-	-	-	-	-
손 씻는 평균 시간	1~5초	5.8	9.1	10.5	7.6	17.7	16.6	19.7	12.9
	6~10초	23.2	24.8	22.2	30.0	33.3	36.7	32.2	39.0
	11~15초	7.8	14.8	8.5	13.6	15.4	15.8	16.2	18.1
	16~20초	14.5	17.2	12.2	17.7	10.3	10.5	8.7	11.0
	21초 이상1)	48.6	34.2	45.9	31.1	18.5	17.9	23.3	19.0
손 씻는 부위2)	손바닥	99.7	91.9	99.6	97.4	99.3	95.6		
	손등	98.4	86.2	96.5	92.3	96.7	93.7		
	손가락	84.9	73.4	85.3	77.1	-	-		
	손가락 사이	79.4	64.6	81.1	64.3	78.9	77.7	-	-
	깍지	73.9	53.3	75.7	58.4	-	-		
	손목	67.1	35.0	70.8	31.0	59.0	68.9		
	손톱 밑	39.9	23.7	43.9	27.3	38.2	35.8		
최근 6개월 동안 질환	감기	25.9	53.1	24.6	52.9	43.0	25.4	45.7	38.1
경험률	식중독	1.8	2.3	2.3	2.1	1.7	1.3	1.5	1.1
	설사	23.1	24.1	25.3	31.9	-	-	-	-
	눈병	4.3	5.1	3.6	8.1	3.4	2.7	3.5	3.9

1)2013년, 2014년 문항의 보기 '21~25초', '26~30초', '31초 이상' 세 항목을 포함.

2) 복수응답 문항임.

가정에서의 손씻기 실태를 상황별로 살펴보면, "화장실 사용 뒤"가 88.0%로 가장 높게 나타났다. 다음으로 "식사 전"(71.9%), "음식 조리 전"(70.2%) 순서로 나타났다. 이는 2013년도에"화장실 사용 뒤"가 87.7%로 가장 높게 나타났으며, 다음으로 "식사 전"(77.5%), "음식 조리 전"(72.5%) 순서로 나타난 것과 유사하다. 2011년도에 "음식 조리 전"가 97.3%로 가장 높게 나타났으며, 다음으로 "화장실 사용 전"(96.2%), "식사 전"(90.3%) 순서로 나타난 것과는 차이를 보였다. 학생들의 가정에서의 손씻기 실태를 살펴보면, "음식 조리 전"가 89.1%로 가장 높게 나타났으며, 다음으로 "화장실 다녀온 후"(86.1%), "식사 전"(58.7%)순서로 나타났다. 2013년도에 "화장실 사용 후"가 87.3%로 가장 높게 나타났으며, 다음으로 "식사 전"(58.7%), "음식 조리 전"(51.7%) 순서로 나타나며 유사했다.

성인들의 일상생활에서 손씻기 실태를 상황별로 살펴보면, "쓰레기를 버린 후"(87.1%), "청소한 후"(80.0%)로 조사되었으며, "돈을 만진 후"(18.1%), "기침/재채기 뒤"(17.6%)로 낮게 나타났다. 이는 2013년도에 "쓰레기를 버린 후"(87.5%), "청소한 후"(80.2%)로 조사되었으며, "돈을 만진 후"

(19.1%), “기침/재채기 뒤”(17.5%)와 유사했다.

학생들의 일상생활에서 손씻기 실태를 상황별로 살펴보면, “청소한 후”(79.5%), “쓰레기를 버린 후”(78.2%)로 조사되었으며, “코풀기나 코를 만진 후”(27.3%), “돈을 만진 후”(24.7%)로 낮게 나타났다. 이는 2013년도에 “청소한 후”(67.2%), “쓰레기를 버린 후”(74.0%)로 조사되었으며, “코풀기나 코를 만진 후”(24.2%), “돈을 만진 후”(24.8%)로 낮게 나타난 것과 유사하였다.

성인들의 외출 시 손씻기는 “공공화장실 사용 뒤” 88.0%로 가장 높게 나타났으며, 이는 2013년도에 “공공화장실 사용 뒤” 89.0%, 2011년도에 “공공화장실 사용 뒤” 93.7%이 가장 높게 나타난 것과 동일하였다. 다음으로 “외출 후 귀가 시”(78.1%), “식당에서 식사 전”(57.1%), “식사 전 물수건 사용”(56.2%) 순서로 나타났다. 학생들의 외출 시 손씻기는 “화장실을 다녀온 후” 86.2%로 가장 높게 나타났으며, 다음으로 “외출 후 귀가 시”(70.6%), “식당에서 식사 전”(60.8%), “식사 전 물수건 사용”(27.0%) 순서로 나타났다.

표 2. 상황별 손씻기 습관 조사 결과 단위: %

조사대상		2014년 성인 19세 이상	2014년 학생 11～18세	2013년 성인 19세 이상	2013년 학생 11～18세	2011년 14세 이상	2009년 14세 이상	2008년 14세 이상	2006년 14세 이상
가정에서[1][2]	식사 전	71.9	58.7	77.5	58.7	90.3	91.9	84.5	85.9
	음식 조리 전	70.2	89.1	72.5	51.7	97.3	98.2	85.3	88.7
	화장실 사용 후	88.0	86.1	87.7	87.3	96.2	96.7	97.3	95.0
	아기돌보기 후	68.1	-	71.2	-	81.7	91.2	87.4	85.6
	기저귀 교체 후	69.4	-	74.5	-	46.7	71.3	75.5	80.0
	애완동물 만진 후	47.3	71.3	59.7	37.3	65.3	75.6	70.1	68.8
일상생활에서[3][4]	돈을 만진 후	18.1	24.7	19.1	24.8	31.3	33.5	28.5	32.2
	기침/재채기 후	17.6	36.4	17.5	19.6	28.7	26.4	24.0	21.1
	코풀기나 코를 만진 후	22.1	27.3	26.1	24.2	-	-	-	-
	쓰레기를 버린 후	87.1	78.2	87.5	74.0				
	청소한 후	80.0	79.5	80.2	67.2				
외출 시[5][6]	식당에서 식사 전	57.1	60.8	61.6	56.7	62.7	87.0	71.7	73.4
	공공화장실 사용 후	88.0	86.2	89.0	83.7	93.7	95.7	91.0	93.3
	외출 후 귀가 시	78.1	70.6	79.8	70.0	92.2	94.8	86.3	86.0
	식사 전 물수건 사용	56.2	27.0	58.3	18.4	-	-	-	-

1) 각 문항의 보기 '항상 씻는 편이다.'와 '대체로 씻는 편이다.'를 포함한 비율을 보여줌.
2) 2013년, 2014년 문항의 보기 '항상 씻었다'와 '자주 씻었다'를 포함한 비율을 보여줌.
3) 각 문항의 보기 '항상 씻는 편이다.'와 '대체로 씻는 편이다.'를 포함한 비율을 보여줌.
4) 2013년, 2014년 문항의 보기 '항상 씻었다'와 '자주 씻었다'를 포함한 비율을 보여줌.
5) 각 문항의 보기 '항상 씻는 편이다.'와 '대체로 씻는 편이다.'를 포함한 비율을 보여줌.
6) 2013년, 2014년 문항의 보기 '항상 씻었다'와 '자주 씻었다'를 포함한 비율을 보여줌.

손씻기가 감염병(전염병) 예방에 어느 정도 도움이 되는지에 대해 조사한 결과, 성인의 경우 '도움이 된다'는 응답은 90.1%로 매우 높게 조사되었다. 학생의 경우에도 성인의 결과와 마찬가지로 '도움이 된다'는 응답은 92.3%로 나타나 거의 대부분의 계층에서 '도움이 된다'는 응답이 90% 이상으로 높게 나타났다. 이는 2013년도 '도움이 된다'는 응답에 성인이 90.6%, 학생이 91.5%로 나타난 것과 유사하다.

한편, 손을 잘 씻지 않는 이유로 '습관이 안 되어서'(31.4%)라는 응답이 가장 높게 나타났으며, 그 다음으로 '씻을 장소가 없어서'(24.7%), '귀찮아서'(23.4%) 순으로 나타났다. 학생의 경우, 그 이유로 '귀찮아서'라는 응답이 35.7%로 가장 높았으며, '습관이 안 되어서'는 22.9%, '씻을 장소가 없어서'는 4.9%로 상당히 낮았다. 이는 2013년도에'습관이

안 되어서'(60.5%)라는 응답이 가장 높게 나타났으며, 그 다음으로 '씻을 장소가 없어서'(6.7%), '귀찮아서'(27.5%) 순으로 나타난 것과 유사하였다. 학생의 경우, 그 이유로 '귀찮아서'라는 응답이 35.7%로 가장 높았으며, '습관이 안 되어서'는 22.9%, '씻을 장소가 없어서'는 4.9%로 상당히 낮았다.

국내외 유행성 질병이 발생했을 때 손 씻기 횟수가 증가하는지에 대해 설문한 결과, 성인은 59.1%가 '그렇다'고 응답하였고, 학생의 경우 '그렇다'(65.2%)는 응답이 '그렇지 않다'(27.2%)는 응답보다 38.0% 높은 비율을 보여, 학생이 성인에 비해 국내외 유행성 질병 발생 시 손씻기 횟수가 증가하는 경우가 상대적으로 많은 것으로 분석됐다.

일반적으로 공중화장실의 손씻기 환경이 잘 되어있는지에 대해, 성인의 경우 '충분하지 않다'는 응답은 29.8%로 나타나 공중화장실 손씻기 환경에 대한 긍정적 평가와 부정적 평가는 비슷한 수준의 양상을 보인다. 2013년도에 성인의 경우 '충분하지 않다'는 응답은 31.4%, 2011년도에 48.2%인 것에 비하여 하락한 수치이다. 반면, 학생의 경우 '충분하지 않다'는 부정적 평가가 45.0%로 더 높게 조사되었으나, 2013년도에 45.3%, 2011년도에 48.2%에 비하여 성인에 비하여 소폭 하락하여 차이를 보였다.

공중화장실에서의 손씻기 활성화를 위한 개선사항으로 성인은 '비누 및 손전용 세제 설치'(33.2%)가 가장 먼저 개선되어야 한다고 응답했으며, 다음으로 '청소 등 깨끗한 환경 유지'(23.3%), '핸드 드라이기나 종이 타월 설치'(16.6%), '세면대 보수 및 개선'(6.6%) 등의 순으로 언급하였다. 학생들의 경우에는 '청소 등 깨끗한 환경 유지'(27.8%)를 가장 먼저 개선되어야 할 사항으로 꼽았으며, 이어 '비누 및 손전용 세제 설치'가 27.1%로 높게 나타났다.

표 3. 손씻기 관련 인식수준 조사 결과 단위: %

		2014년		2013년		2011년	2009년	2008년	2006년
		성인	학생	성인	학생				
조사대상		19세 이상	11~18세	19세 이상	11~18세	14세 이상	14세 이상	14세 이상	14세 이상
손씻기의 질병 예방 효과 긍정적 인식 정도[1]		90.1	92.3	90.6	91.5	84.0	87.0	79.0	86.3
본인의 손씻기 횟수에 대한 부정적 자가 평가의	귀찮아서	23.4	35.7	27.5	71.2	29.2	33.7	29.5	47.1
	씻을 장소가 없어서	24.7	4.9	6.7	1.5	5.9	7.2	6.8	8.8
	습관이 안 되어서	31.4	22.9	60.5	25.8	59.7	45.2	54.5	41.2

손을 잘 씻지 않는 이유	기타	2.4	13.8	5.3	1.5	5.2	8.9	9.1	2.9
국내외 유행성 질병 발생 시 손씻기 횟수 증가 여부		59.1	65.2	-	-	-	-	-	-
공공화장실의 손씻기환경에 대한 부정적 평가[2) 3)]		29.8	45.0	31.4	45.3	48.2	49.7	46.0	47.1
손씻기 활성화를 위한 공중화장실 개선 사항	비누 및 세제 설치	33.2	27.1	34.5	30.1	-	-	-	-
	깨끗한 환경 유지	23.3	27.8	28.4	39.7				
	핸드 드라이기/종이타월	16.6	12.8	15.8	13.3				
	세면대 보수 및 개선	6.6	7.3	8.3	10.1				
	교육 및 홍보 강화(안내문 부착)	3.5	1.6	4.7	2.0				
	온수기 설치	2.3	5.0	2.5	4.4				

1) 각 문항의 보기 '매우 도움이 된다.'와 '도움이 되는 편이다.'를 포함한 비율을 보여줌.

2) 2009년, 2011년 문항의 보기 '매우 부족 ', '부족한 편' 두 항목을 포함.

3) 2013년, 2014년 문항의 '전혀 충분하지 않다'와 '충분하지 않다' 두 항목을 포함.

최근 1년 동안 올바른 손씻기 교육을 받은 경험이 있는지에 대해 조사한 결과, 성인남녀의 10명 중 약 1명(12.9%) 정도만 손씻기 교육 경험이 있었으며, 학생들의 경우, 10명 중 약 4명 정도(40.6%)가 교육 경험이 있는 것으로 조사되었다. 이는 2013년도에 만19세 이상 성인의 경우 12.8%에 비하여 소폭 상승하였으나, 학생들의 경우 41.6%에 비하여 하락한 수치이다.

손씻기 교육을 받은 경험이 있는 성인의 경우(N=647) 최근 1년간 교육받은 횟수는 평균 '2.5회'로 조사되었으며, 세부 빈도별로는 '1회'가 47.5%로 가장 높았으며, '4회'(3.6%)는 상대적으로 낮은 수준을 보인다. 2013년도에 조사된 세부 빈도별로는 '1회'가 57.0%로 가장 높았으며, '4회'(1.6%)는 상대적으로 낮은 수준이었던 것과 유사하다. 손씻기 교육을 받은 경험이 있는 학생의 경우(N=365) 최근 1년간 평균 '1.7회' 교육을 받은 것으로 나타났으며, 세부 빈도별로 '1회' 받았다는 응답이 57.0%로 가장 높게 나타났다. 2013년도 조사된 학생의 경우, 세부 빈도별로 '1회' 받았다는 응답이 54.6%로 가장 높았던 것과 동일하였다. 손씻기 교육 경험이 있는 성인(N=647)의 경우, 평균 1회 교육 시간으로 '10분미만'이라는 응답이 51.3%로 가장 많았으며, 손씻기 교육 경험이 있는 학생(N=365)의 경우에는 평균 1회 교육 시간으로 '30분~1시간미만'이라는 응답이 43.6%로 가장 높게 나타났다. 다음으로 '10~30분미만'(34.8%)로 높게 나타났다. 이는 2013년도에 손씻기

교육 경험이 있는 성인의 경우, 평균 1회 교육 시간으로 ‘10분미만’이라는 응답이 46.6%로 가장 높았던 것으로 동일하였고 손씻기 교육 경험이 있는 학생의 경우에는 평균 1회 교육 시간으로 ‘10분~30분 미만’이라는 응답이 40.5%로 가장 높게나온 것과는 차이를 보였다.

손씻기 교육을 받은 성인(N=647)이 교육을 받은 방법으로 ‘강의’를 통해서 교육을 받았다는 응답이 40.0%로 가장 높았으며, 다음으로 ‘비디오 등 시청각 자료’(30.0%), ‘가정통신문 등의 유인물’(16.8%), ‘실습’(12.7%)의 순서로 나타났다. 이는 2013년도에 손씻기 교육을 받은 성인이 교육을 받은 방법으로 ‘강의’를 통해서 교육을 받았다는 응답이 43.0%로 가장 높았던 것과 동일하다. 손씻기 교육 경험이 있는 학생(N=365)의 경우 ‘시청각 자료’(38.4%)와 ‘강의’(29.0%)를 통해 손씻기 교육을 받았다는 응답이 높게 조사되었으며, ‘실습’을 통한 교육은 8.2%로 미미한 수준이다. 이는 2013년도에 손씻기 교육 경험이 있는 학생의 경우 ‘강의’(35.7%)로 가장 높았던 것과 차이를 보인다.

성인은 손씻기 교육 필요성에 대해 ‘필요하다’라는 응답은 80.6%로 높았고, 학생의 경우 77.7%가 손씻기 교육이 ‘필요하다’고 응답하였다. 이는 2013년도에 성인은 손씻기 교육 필요성에 대해 ‘필요하다’라는 응답은 95.2%로 높았고, 학생의 경우 93.2%가 손씻기 교육이 ‘필요하다’고 응답한 것에 비해 하락한 수치이다.

최근 1년 동안 올바른 손씻기 관련 광고 및 홍보물을 본 적이 있는지에 대해, 성인의 절반 이상(57.1%)이 경험이 있다고 응답하였고, 42.9%는 경험이 없는 것으로 조사되었다. 이는 2013년도에 성인의 절반 이상(56.4%)이 경험이 있다고 응답하였고, 43.6%는 경험이 없는 것으로 조사되 것과 유사하였다. 학생의 경우, 올바른 손씻기 광고 및 홍보 경험에 대해 ‘경험 있음’이라는 응답은 56.6%로 조사된 반면, ‘경험 없음’은 43.4%로 나타나 성인의 결과와 비슷한 양상을 보인다. 손씻기 관련 광고 및 홍보물을 접한 경험이 있는 성인(N=2,863)을 대상으로 어느 매체를 통해 접했는지 설문한 결과, ‘지상파TV’가 47.7%로 가장 높았으며, 이어 ‘보건소 및 병의원 PDP’가 31.9%로 나타났다. ‘옥외전광판’(7.2%), ‘인터넷’(7.8%), ‘버스’(4.8%) 등의 매체는 10% 미만으로 미미한 수준을 보인다. 이는 2013년도에 성인의 경우 손씻기 관련 광고 및 홍보물을 접한 경험 매체에 대

하여 ‘지상파TV’가 47.0%로 가장 높았던 것과 동일하다. 학생(N=509)의 경우, 어느 매체를 통해 접했는지 설문한 결과, ‘보건소 및 병의원 PDP’가 37.7%로 가장 높았으며, 이어 ‘지상파TV’가 37.5%로 나타났다. ‘인터넷’(16.7%), ‘버스’(9.6%) 등의 매체 순으로 나타났다.

손씻기 관련 광고 및 홍보물의 내용이 이해하기 쉬운지에 대한 조사 결과, 광고 및 홍보를 접한 경험이 있는 성인(N=2,863)의 94.1%는 ‘이해하기 쉽다’고 응답하였으며, 3.5%는 ‘이해하기 쉽지 않다’고 응답한다. 이는 2013년도에 광고 및 홍보를 접한 경험이 있는 성인의 94.5%는 ‘이해하기 쉽다’고 응답하였으며, 5.5%는 ‘이해하기 쉽지 않다’고 응답한 것과 유사하였다. 손씻기 광고 및 홍보물 경험이 있는 학생(N=509)의 경우, ‘이해하기 쉽다’는 응답은 91.4%로 상당히 높은 수준을 보였으며, ‘이해하기 쉽지 않다’는 응답은 6.1%로 조사되었다. 손씻기 광고 및 홍보물을 본 후, 감염병과 관련된 손씻기 등 생활태도를 바꿔야겠다는 생각이 들었는지에 대해, 손씻기 광고 및 홍보 경험이 있는 성인(N=2,863)의 84.0%는 ‘그렇다’고 응답하였다. 이는 2013년도에 성인의 83.5%가 ‘그렇다’고 응답한 것에 비하여 소폭 상승한 수치이다. 손씻기 광고 및 홍보 경험이 있는 학생(N=509)의 경우, 10명 중 약 8명(76.2%)은 ‘손씻기 등 생활태도를 바꿔야겠다’고 응답하였다.

손씻기 광고 및 홍보물을 본 후, 손씻기 방법에 대해 제대로 알게 되었는지에 대한 조사 결과, 손씻기 광고 및 홍보 경험이 있는 성인(N=2,863)의 90.2%가 ‘알게 되었다’고 답한 반면, 7.8%는 ‘그렇지 않다’고 응답하였다. 이는 2013년도에 비해 손씻기 광고 및 홍보물을 본 후, 손씻기 방법에 대해 제대로 알게 되었는지에 대한 조사에 대하여 성인의 88.5%가 알게 되었다’고 답한 것에 비하여 상승한 수치이다. 손씻기 광고 및 홍보 경험이 있는 학생(N=509)의 경우, 10명 중 약 9명(87.6%)이 손씻기 방법에 대해 제대로 알게 되었다고 응답하였으며, 학생의 9.6%는 ‘그렇지 않다’고 응답했다.

표 4. 손씻기 관련 교육 및 광고/홍보에 대한 평가 조사 결과 단위: %

	2014년		2013년		2011년	2009년	2008년	2006년
	성인	학생	성인	학생				
조사대상	19세 이상	11～18세	19세 이상	11～18세	14세 이상	14세 이상	14세 이상	14세 이상
손씻기 교육 및 홍보물 유 경험 응답	-	-	-	-	57.2	48.5	27.2	17.4

률									
손씻기 교육 경험률		12.9	40.6	12.8	41.6	-	-	-	-
자녀에 대한 손씻기 교육 실시 경험 응답률		54.4	-	71.0	-	58.9	66.8	70.4	66.8
부모님으로부터 손씻기 교육 경험률		-	50.4	-	-	-	-	-	-
최근 1년간 교육 받은 횟수	1회	47.5	57.0	45.6	54.6				
	2회	26.3	30.4	33.8	23.0				
	3회	11.7	6.6	7.3	13.4	-	-	-	-
	4회	3.6	1.6	6.6	1.7				
	5회 이상	11.0	4.4	6.8	7.2				
평균 1회 교육 시간	10분 미만	51.3	13.7	46.6	18.2				
	10분-30분	30.7	34.8	33.3	40.5	-	-	-	-
	30분-1시간	11.1	43.6	12.7	37.8				
	1시간 이상	5.2	5.5	6.0	3.4				
손씻기 교육 방법	강의	40.0	29.0	43.0	35.7				
	시청각 자료	30.0	38.4	35.6	32.6	-	-	-	-
	유인물	16.8	23.0	12.7	24.7				
	실습	12.7	8.2	8.7	6.5				
손씻기 교육 필요성에 대한 필요성 인식[1]		80.6	77.7	95.2	93.2	-	-	-	-
올바른 손씻기 관련 광고 및 홍보 경험		57.1	56.6	56.4	56.1	-	-	-	-
광고 전달 매체	지상파 TV	47.7	37.5	47.0	46.8				
	보건소 병의원 PDP	31.9	37.7	29.0	41.2				
	인터넷	7.8	16.7	8.2	18.6	-	-	-	-
	버스	4.8	9.6	3.6	15.8				
	지하철	10.4	6.5	8.0	10.7				
	케이블TV	5.5	7.7	4.6	6.4				
	옥외전광판	7.2	8.1	9.9	6.1				
손씻기 광고 및 홍보물의 이해도[2]		94.1	91.4	94.5	95.2	-	-	-	-
광고 및 홍보 경험 후 손씻기 등 생활태도 변화인식 여부[3]		84.0	76.2	83.5	80.1	-	-	-	-
광고 및 홍보 경험 후 손씻기 방법 숙지 여부[4]		90.2	87.6	88.5	90.1	-	-	-	-

[1] '매우 그렇다.'와 '대체로 그렇다'를 포함.

[2] '매우 그렇다.'와 '대체로 그렇다'를 포함.

[3] '매우 그렇다.'와 '대체로 그렇다'를 포함.

[4] '매우 그렇다.'와 '대체로 그렇다'를 포함.

2. 관찰조사

화장실에서 용변 후 비누로 손을 씻는 비율은 29.5%로 나타났으며, 2013년(23.5%)에 비해 6.0% 증가, 2011년(19.1%)에 비해 10.4% 증가한 것으로 조사되었다. 신종 플루가 유행하던 2009년이 가장 높았다. 2013년도보다 손을 씻는 사람은 감소하였으나, 비누를 이용하여 손을 씻은 사람은 증가하였다. 화장실에서 용변 후 손을 씻는 비율은 71.4%로 나타났으며, 2011년(57.5%)에 비해 15.1% 증가, 2013년(72.6%)에 비해 1.2% 감소한 것으로 조사되었다. 계층별로 살펴보면, 손을 씻는 경우는 남성(66.2%)보다 여성(76.6%)에서 상대적으로 높은 특징을 보인다. 이는 2013년도 남성(63.8%), 여성(81.4%)와 2011년도 남성(45.9%), 여성(69.2%)와 동일하게 나타났다. 화장실에서 용변 후 손을 씻은 사람 중 비누를 사용하는 이용자는 41.2%로 지난 2013년(32.3%), 2011년(33.2%)결과에 비해 증가하였다.

손 씻는 시간을 측정 결과, 1~5초(39.0%), 6~10초(33.1%), 11~15초(12.9%), 16~20초(7.4%), 21초 이상(7.6%) 순으로 나타났다. 손 씻는 시간을 분석해 보면, 평균 약 9초 정도로 2013년(약 8초)에 비해 다소 증가하였다.

손을 씻고 난 후 수도꼭지를 잠그는 방법으로는 화장실 이용자의 대부분(99.4%)이 '맨손'으로 잠그는 것으로 관찰되었나, 휴지를 이용하여 잠그는 이용자(0.1%)도 일부 존재하였다. 손을 씻은 후 물기 제거 방법으로는 '핸드 드라이어 사용'이 23.4%로 가장 많았으며, 다음으로 '화장실 비치 휴지(종이타월) 사용'(16.3%), '개인용 손수건 사용'(2.9%) 순으로 나타났다.

표 5. 손씻기 관찰조사 결과 단위: %

Year		2014	2013	2011	2009	2008	2006
공공화장실 이용 후 비누로 손씻기율 (Handwashing with soap after using public restrooms)		29.5	23.5	19.1	35.6	18.6	17.0
손 씻은 이 중 비누사용율		41.3	32.3	33.2	47.6	31.1	30.0
공공화장실 이용 후 손씻기율 (Handwashing after using public restrooms)		71.4	72.6	57.5	74.7	60.1	63.5
성별	남성	66.2	63.8	45.9	64.7	53.6	-
	여성	76.6	81.4	69.2	84.8	66.5	
연령	10대	68.2	67.3	53.7	67.0	65.4	-
	20대	76.7	75.9	65.9	80.4	64.7	
	30대	74.5	76.4	60.5	74.	63.4	
	40대	73.6	72.2	52.9	72.9	52.5	
	50대 이상	62.7	64.9	40.7	65.5	54.0	
지역	서울	78.8	68.3	51.0	80.8	57.9	-
	인천	73.1	71.7	57.3	80.6	50.7	
	부산	63.1	67.5	46.6	63.1	61.2	
	대구	68.1	75.0	56.8	73.6	65.1	
	광주	62.5	75.8	50.4	70.0	67.1	
	대전	79.4	75.0	57.3	76.9	57.2	
	울산	75.0	75.0	83.5	78.1	61.2	
손 씻는 평균 시간	1~5초	39.0	46.6	39.3	27.7	40.5	45.4
	6~10초	33.1	29.0	28.8	28.2	32.9	31.6
	11~15초	12.9	12.1	17.8	19.7	16.9	13.6
	16~20초	7.4	7.5	8.8	14.9	6.6	5.4
	21초 이상	7.6	4.8	5.3	9.5	3.1	3.9
수도꼭지 잠그는 방법	맨손으로 잠금	99.4	99.2	95.7	98.8	43.8	58.2
	종이타월 사용	0.1	-	3.2	0.5	0.2	-
	자동[2)]	0.3	0.8	1.1	0.7	56.0	41.8
손 씻은 후 물기제거방법	종이타월	16.3	14.1	20.4	22.2	49.9	54.3
	공용수건	-	-	-	-	0.2	-
	핸드 드라이어	23.4	23.9	30.4	30.0	25.8	15.7
	개인 손수건	2.9	3.1	3.5	3.4	1.4	2.8
	물기제거안함[3)]	52.0	57.9	41.0	42.4	22.7	27.1

[1] 2009년, 2011년 행태조사에서는 10대 보기를 10대 이하로 대체함.

[2] 2009년, 2011년 행태조사에서는 자동 보기를 수도꼭지 잠그지 않음으로 대체함.

[3] 2006년, 2008년 행태조사에서는 물기제거안함을 그냥 말림/기타로 대체함.

IV. Discussions

비누로 손씻기는 감염병 예방을 가장 기초적이고 경제적인 방법으로 수인성 및 식품매개감염병의 약 50~70%, 폐렴, 농가진, 설사질환의 40~50% 이상을 예방하며, 특히 지구온난화와 더불어 출현 또는 재출현하는 사스, 인플루엔자, 감기, 콜레라, 이질, 유행성 눈병 등 대부분의 감염병이 비누로 손씻기로 예방 가능하다.

현재 손씻기가 감염병(전염병) 예방에 어느 정도 도움이 되는지에 대해 조사한 결과 '도움이 된다'는 응답이 80% 내외로 높게 나타났다. 국민들은 대부분 손씻기의 예방 효과를 인지하고 있으나 실천이 미비한 실정이다. 또한, 인식도조사에 따르면 화장실 사용 후 비누 사용율이 75% 내외로 조사되었으나 관찰조사 결과를 살펴보면 용무 후 비누로 손을 씻는 이용자는 29.5%로 뿐이었다. 대부분 손씻기의 예방효과와 본인의 손씻기 횟수에 대한 긍정적 자가 평가 비율은 높게 조사되지만 관찰조사는 부족한 결과를 보인다. 지속적인 실태조사는 국민들의 손씻기 지식과 태도, 실천에 대한 정보를 제공해 줄 것이며 이는 손씻기 사업에 대한 근거로 이용될 수 있다.

전 세계적 손씻기 행위와 건강 효과 업데이트에 대한 42개 문헌 연구에 따르면, 전세계 인구의 19%의 사람만이 배변 후 (예를 들면, 위생 시설을 사용하거나 어린이의 배설물에 접촉했을 때) 비누로 손을 씻는 다는 것을 추정하였다. 지역별 배변 후 비누로 손씻기율은 저개발 및 중진국에선 13%에서 17%, 선진국에선 42%에서 49%정도로 추정하였다. 국가별 배변 후 비누로 손씻기율은 뉴질랜드가 72%로 가장 높았으며 영국(52%), 네덜란드(50%), 미국(49%)순으로 높게 나타났다. 2014년도 우리나라 배변 후 비누로 손씻기율은 29.5%로 태국(25%), 에티오피아(22%)와 유사하게 추정되었고 부르키나파소의 비누로 손씻기율은 8%이며, 탄자니아의 비누로 손씻기율은 5%로 가장 낮았다. 손씻기의 긍정적 건강 효과에도 불구하고, 배변 후 손씻기가 세계적으로 제대로 실행되고 있지 않다는 것을 보여주고 있다.

현재 손씻기 사업은 질병관리본부와 범국민손씻기운동본부, 서울시 및 각 광역단체, 그리고 식품의약품안전처(청)에서 각각 개별 기관의 목적에 따라 추진되고 있으나 이에 대한 효과 평가 및 타당도에 대한 평가가 없으며, 실태조사를 수행하는 기관은 범국민손씻기운동본부만 존재하였고 현재 중단되었다. 범국민손씻기운동본부의 손씻기 실태

조사는 조사문항의 표준화(용어의 표준, 조사문항의 내용 및 범위 등), 조사의 기획성, 목적성 등이 불명확하며, 전반적으로 체계화되어 있지 않다. 또한 연도별 주기 및 시점이 일정치 않으며, 조사시점은 조사결과에 대하여 많은 변이를 줄 수 있음에도 불구하고 모두 다르게 조사되었으며, 사례 조사 수 또한 차이가 있었다. 수년 동안 조사문항 등의 변화(추소, 확대, 수정, 추가 등) 없이 단순히 전국규모의 간단한 손씻기 관련 통계를 생산하는 수준으로 판단되며 결과와 사업간 연계 또는 연동되거나 환류, 반영되는 프로그램이 없어 개선이 필요한 부분이다.

비누로 손씻기 사업 또는 프로그램은 손씻기 지식과 태도를 기르는 구성이어야 하며 이를 뒷받침해 줄 수 있는 체계적인 실태조사가 필요하다. 실태조사를 통하여 손씻기 수준 모니터링 및 평가, 홍보사업 평가 등 현재 수행한 사업 또는 프로그램의 효과를 알아 볼 수 있으며 사업의 개선점, 국민들의 요구 등을 파악할 수 있다. 이러한 정보를 바탕으로 비누로 손씻기 캠페인을 지속적으로 시행하여야한다. 비누로 손씻기 캠페인은 사회적으로 주목을 받고, 국민 개개인에게 보다 접근성 있게 운영되기 위해 통합 브랜드 캠페인으로 개발되어야 한다. 또한, 국내의 환경적 요인과 대상자의 이해정도, 그리고 프로그램의 효율적 운영 등 캠페인 기획 배경과 방향에 고려되어야 하는 상황들을 우선적으로 고려하여, 이를 위한 정책사업도 준비하여야 한다. 정책사업의 프레임은 사회적 마케팅 활동에 고려해볼 수 있는 요소들을 포함하고 있으며, 전체적인 홍보 전략과 방향에 적합한 프레임으로 구성되어 있어야한다.

세계보건기구(WHO)와 비누로 손씻기 세계 공공-민간 파트너십(The Global Public -Private Partnership for Handwashing with Soap, PPPHW)은 손씻기와 어린이 건강에 초점을 맞춘 세계적인 홍보 캠페인 사업 등을 추진하고 있으며 이러한 흐름에 맞도록 각국에서 사업을 진행하고 있으며, 매년 유행과 흐름에 맞추어 손씻기 행사를 진행하고 있었다. 하지만, 현재 우리나라의 손씻기 사업은 사업에 대한 이해가 부족하고, 사업의 운영관리체계가 없음으로 인하여 전략적, 체계적 운영관리가 시급히 요구되며 손씻기 사업 추진전략 틀을 제시한다.

비누로 손씻기 사업은 이를 국가의 중요한 공중보건문제로 인식하고 보건의료사업에서 기본적이고, 우선순위 사업에 설정토록 해야 하며 이를 위하여 공공 및 민간영역, 관련 부처 및 기관과의 협력체계를 구축한다. 체계적인 손씻기 실태조사 체계를 구축하여 비누로 손씻기 사업의 확대추진을 위한 과학적 근거를 확보하고 근거 기반의 효율적인 정책을 개발·실행하기 위한 연구 개발

을 지원하는 전략 틀이 필요하다.

이 연구의 제한점은 첫째, 2013년도 이전에 시행된 조사는 조사시기가 불규칙적이며, 2013년도 이후 조사와 조사 설계에 차이점이 있다. 조사대상, 표본크기, 조사지점 등 조사 설계에 차이가 있어 비교에 어려움이 있다. 둘째, 예산에 맞추어 설계된 조사로 표본크기가 충분하지 못하여 대표성에 문제가 존재한다. 셋째, 관찰조사를 통해 조사된 화장실 사용 후 비누로 손씻기를 통해 국가간 비교를 실시하고 싶었으나, 국가 간 비교지표가 혼재되어 있어 비교하기에 부족한 부분이 존재한다.

그럼에도 불구하고 이 연구는 현재 국가차원에서의 손씻기율을 모니터링 평가하고 광역시도별 모니터링 및 평가하고, 광역시도별 모니터링 및 평가가 가능한 기초자료를 제공하여 손씻기 사업을 진단하고 개선하고자 하는 목적으로 활용되는 바에 의의가 있다.

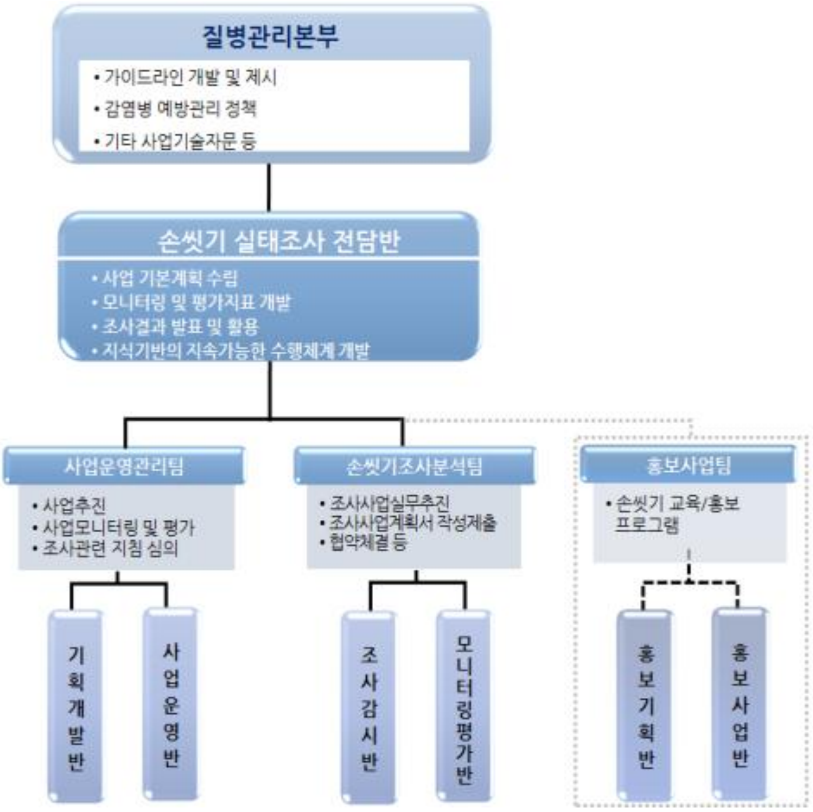

그림 1. 손씻기 실태조사 수행체계(안)

References

1. UNICEF Malaysia Communications. 2002. Global Handwashing Day Planner's Guide, Frequently Asked Questions.
2. Park KJ. 2001. The Development of Estimate Model of Social Economic Costs and Estimates Socioeconomic Effect of Foodborne Disease in Korea. Seoul: Korea Health Industry Development Institute. (Korean).
3. Korea Center for Disease Control. Laboratory reports of bacterial isolation from gastrointestinal infections by province, May 2006. Communicable Diseases Monthly Report 2006;17(6):20. (Korean).
4. Intergovernmental Panel on Climate Change. 2007. 지구온난화보고서.
5. Park JH, Cheong HK, Son DY, Kim SU, Ha CM. 2010. Perceptions and behaviors related to hand hygiene for the prevention of H1N1 influenza transmission among Korean university students during the peak pandemic period. BMC Infectious Diseases. 10:222:1-8.
6. Sheree MS Smith. 2009. A review of hand-washing techniques in primary care and community settings, Journal of Clinical Nursing. 18: 786–90.
7. Luby SP, Agboatwalla M, Feikin DR, Painter J, Billhimer W, Altaf A, Hoekstra RM. 2005. Effect of handwashing on child health: A randomized controlled trial. Lancet. 366(9481): 225-233
8. Ejemot RI, Ehiri JE, Meremikwu MM, Critchley JA. 2008. Hand washing for preventing diarrhoea. Cochrane Database Syst Rev. Jan 23;(1):CD004265.
9. Rabie T and Curtis V. 2006. Handwashing and risk of respiratory infections: a quantitative systematic review. Trop Med Int Health. Mar. 11(3):258-67.
10. Boyce JM, Pittet D. 2002. Healthcare Infection Control Practices Advisory Committee; HICPAC/SHEA/APIC/IDSA Hand Hygiene Task Force. Guideline for Hand Hygiene in Health-

Care Settings. Recommendations of the Healthcare Infection Control Practices Advisory Committee and the HICPAC/SHEA/APIC /IDSA Hand Hygiene Task Force. Society for Healthcare Epidemiology of America/Association for Professionals in Infection Control/ Infectious Diseases Society of America. MMWR Recomm Rep. 51(RR-16):1-45

11. Curtis V, Caimcross S. 2003. Effect of washing hands with soap on diarrhoea risk in the community: A systematic review. Lancet Infect Dis. 3(5):275-281

12. Aledort JE, Lurie N, Wasserman J, Bozzette SA. 2007. Non-pharmaceutical public health interventions for pandemic influenza: an evaluation of the evidence base. BMC Public Health 7:208.

13. Burton M, Cobb E, Donachie P, Judah G, Curtis V, Schmidt WP. 2011. The effect of handwashing with water or soap on bacterial contamination of hands. Int J Environ Res Public Health. Jan;8(1):97-104.

14. Aiello AE, Coulborn RM, Perez V, Larson EL. 2008. Effect of Hand Hygiene on Infectious Disease Risk in the Community Setting: A Meta-Analysis. Am J Public Health. 98:1372-1381.

15. Pittet D, Boyce JM. 2001. Hand hygiene and patient care: pursuing the Semmelweis legacy. Lancet Infect Dis. April: 9–0.

16. Pittet D, Hugonnet S, Harbarth S, Mourouga P, Sauvan V, Touveneau S, Perneger TV. 2000. Effectiveness of a hospital-wide programme to improve compliance with hand-hygiene. Lancet. 356: 1307–2.

17. Pittet D. 2005. Clean hands reduce the burden of disease, Lancet. 366: 185-18

18. 현대리서치. 2006년도 손씻기 실태 및 대국민 인식도 조사 결과보고서. 질병관리본부. 2006.

19. 현대리서치. 2008년도 손씻기 실태 및 대국민 인식도 조사 결과보고서. 질병관리본부. 2008.

20. 코리아리서치. 2009년도 손씻기 실태 및 대국민 인식도 조사 결과보고서. 질병관리본부. 2009.

21. 코리아리서치. 2011년도 손씻기 실태 및 대국민 인식도 조사 결과보고서. 질병관리본부. 2011.

22. 건양대학교 의과대학 예방의학교실. 2013년도 감염병예방을 위한 손씻기 실태조사 및 실천률 향상 전략 개발 . 질병관리본부. 2013년 12월
23. 건양대학교 의과대학 예방의학교실. 2014년도 손씻기 실태조사 결과보고서. 질병관리본부. 2015년 1월
